# Finite element analysis-assisted surgical planning and evaluation of flap design in hand surgery

**DOI:** 10.3389/fbioe.2025.1611993

**Published:** 2025-06-11

**Authors:** Guang Yang, Hui Shen, Yewon Jang, Xiangyi Cheng

**Affiliations:** ^1^ Department of Hand and Foot Surgery, China-Japan Union Hospital of Jilin University, Changchun, Jilin, China; ^2^ Dr. Carl D. Clay and H. Jane Clay Department of Mechanical Engineering, The T.J. Smull College of Engineering, Ohio Northern University, Ada, OH, United States; ^3^ Department of Mechanical Engineering, Frank R. Seaver College of Science and Engineering, Loyola Marymount University, Los Angeles, CA, United States

**Keywords:** finite element analysis, finite element method, flap design, hand surgery, surgical planning, surgical evaluation, mixed reality, augmented reality

## Abstract

Given the anatomical variability among patients and the intricate geometry of the hand, the shape and size of the skin flap have traditionally relied heavily on the surgeon’s experience and subjective judgment. This dependence can lead to inconsistent and sometimes suboptimal results, particularly in complex cases such as web reconstruction in syndactyly surgery. Finite element analysis (FEA) provides a quantitative method to simulate and optimize skin flap design during surgery. However, existing FEA studies in this field are scattered across a wide range of seemingly unrelated topics. To address this, we present a comprehensive review focused on the application of FEA in skin flap design since 2000, with attention to all aspects of preprocessing and postprocessing. The primary objective is to evaluate the potential of FEA to generate patient-specific models by integrating individualized anatomical and biomechanical data while identifying key advancements, analyzing methodological challenges, exploring emerging technologies, and outlining future research directions. A critical finding is that the mechanical modeling of skin remains a major limitation in current FEA applications. To address this, future studies should focus on the development and refinement of non-invasive techniques for acquiring patient-specific skin properties. We also recommend several additional research directions based on our findings. These include exploring techniques to unfold 3D wound surfaces into 2D representations, which can improve mesh quality and computational efficiency; validating FEA simulations through large-scale, multicenter clinical studies to ensure robustness and generalizability; developing real-time AR/MR systems that integrate simulation or optimization results into surgical workflows; and creating AI-powered platforms that learn from clinical data to provide adaptive and personalized flap design recommendations. These findings offer a pathway to bridge the gap between simulation and clinical practice, ultimately aiming to improve surgical outcomes.

## 1 Introduction

**FIGURE 1:**
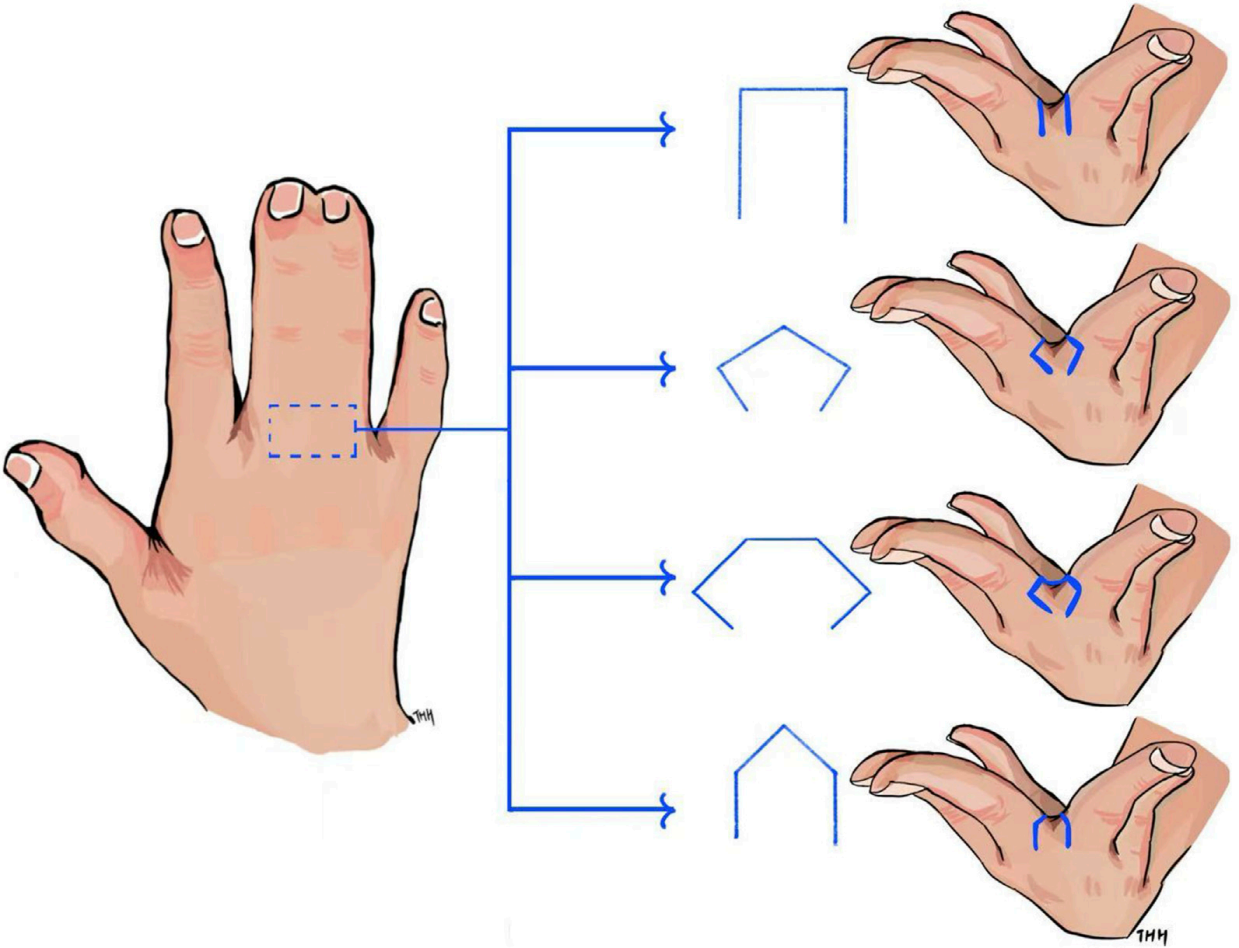
Different flap designs in hand reconstructive surgery.

In the modern industrial world, engineers diligently work to standardize and quantify processes in product design, optimization, and manufacturing. Subjective judgment should be minimized as much as possible to ensure consistency, reliability, and repeatability in practice. This principle has been highly effective. However, surgery in the medical field, which involves the human body and living tissues, presents a different challenge. The complexity and variability of individual human anatomy, such as tissue characteristics, make it difficult to precisely quantify surgical procedures—particularly in hand skin flap design. For instance, syndactyly, one of the most common congenital hand malformations, results from the incomplete separation of adjacent digits. Reconstructive surgery is required to divide the fused skin, reconstruct the web space between fingers, and cover the separated digits with soft tissues (Braun et al., 2016; Wang et al., 2020). Among the critical steps in this procedure, reconstructing the web space is the most essential, requiring the use of a skin flap for commissure reconstruction. Various skin flap designs have been developed for reconstruction, including rectangular (Braun et al., 2016), hexagonal (Wang et al., 2020), pentagonal (Gao et al., 2011), and omega-shaped (D’arcangelo et al., 1996) flaps, as shown in Figure 1. The shape and size of the skin flap directly influence surgical outcomes of the reconstructed web, including aesthetics and functionality. However, despite decades of performing numerous flaps for syndactyly, there is still no consensus on the optimal flap design. Skin flap selection in hand surgeries remains largely dependent on the surgeon’s preference, training, experience, and subjective judgment (Braun et al., 2016). This reliance on individual expertise can lead to suboptimal patient outcomes, particularly when designing flaps for complex, non-flat surfaces where subjective estimations may fall short. Inaccurate estimations may result in excessive or insufficient use of the skin flap, leading to inappropriate distribution of stress and strain within the flap. This distribution affects healing outcomes, including independent digit mobility and the risk of web creep (Gao et al., 2011). As noted by Ogawa et al. (2012), excessive post-surgical tension in a skin flap due to insufficient design can even result in flap necrosis.

To explore skin flap design and identify optimal approaches, researchers have conducted numerous studies in this field. Due to the high costs and inherent limitations of experimental methods on live human tissues, along with advancements in computer technology, they have increasingly turned to finite element analysis (FEA), also known as the finite element method (FEM), to quantify and optimize skin flap surgery. After reviewing the findings in the field, we found that the existing FEA studies appear to take various approaches and examine a variety of seemingly unrelated topics in skin flap surgery. Some researchers examined scar patterns (Miyamoto et al., 2010), while others analyzed the effect of material parameters on stress and strain in flap design (Remache et al., 2015; Ji et al., 2024a). Additionally, some studies explored how the geometric configuration of the skin flap influences the final stress distribution (Pauchot et al., 2013; Lee et al., 2019; Chen et al., 2025). Therefore, it is essential to analyze the available information to understand current research interests and general methodologies, identify the challenges in its application, and determine a direction for future modeling work.

This review aims to evaluate the potential of FEA to generate patient-specific models by integrating individualized anatomical and biomechanical data while also identifying key advancements, analyzing methodological challenges, exploring emerging technologies, and outlining future research directions. It explores the feasibility of applying the numerical method (i.e., FEA) in surgical planning and flap design evaluation, considering individualized material parameters and geometry. Given the similarities in flap design between hand surgery and other plastic surgery procedures, relevant FEA studies from plastic surgery that could be applied to hand skin flap design are also included. The review also explores the potential of combining this numerical analysis with novel technology platforms, such as mixed reality (MR) and augmented reality (AR), to create an interactive demonstration of the surgical process, from pre-surgery design to post-surgery prediction. This review is intended to benefit both researchers and clinical end-users.

## 2 Methodology

**FIGURE 2:**
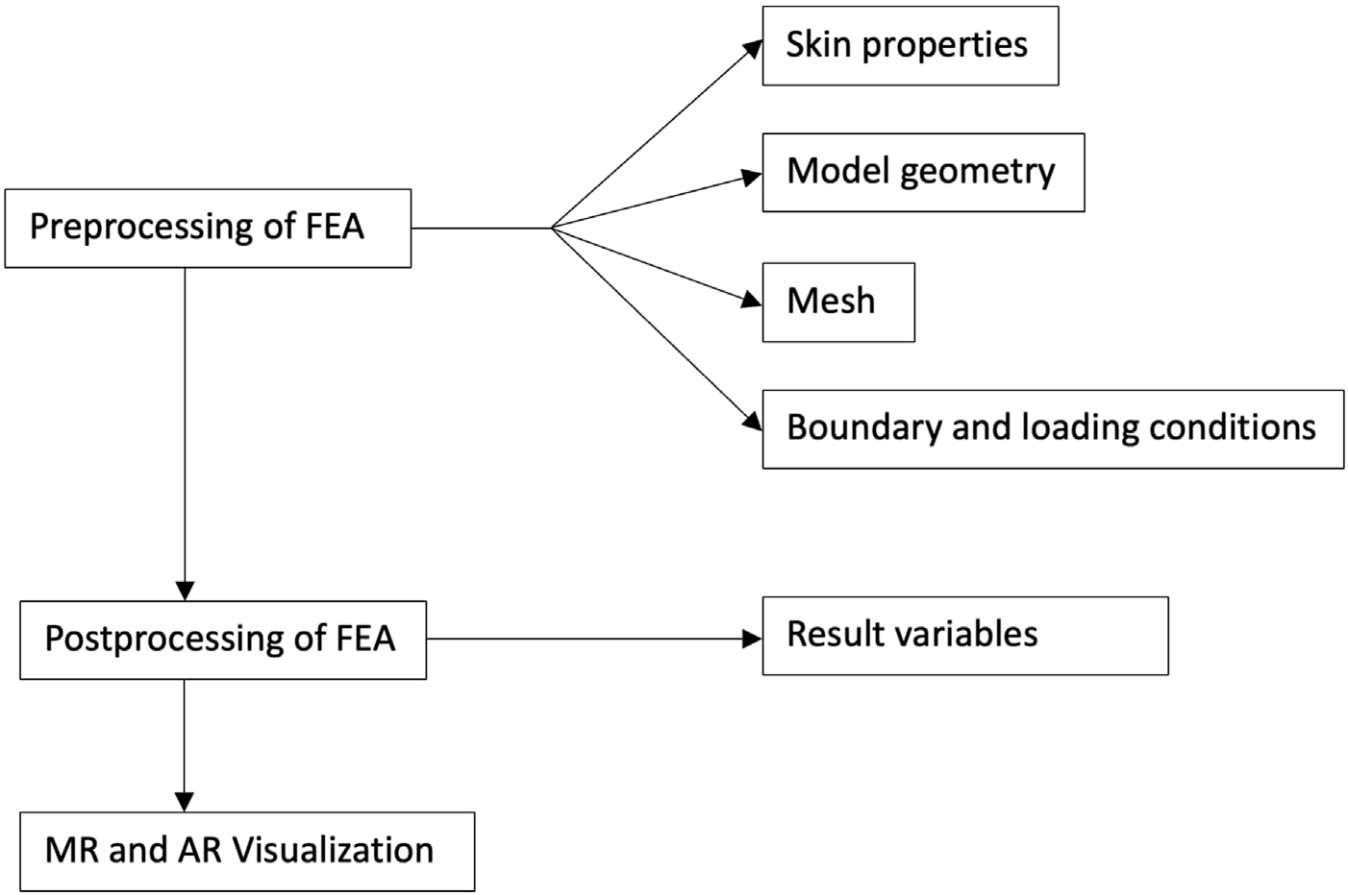
Overview of the workflow for this review.

In the FEA of skin flaps, several preprocessing decisions must be made, including defining tissue properties (e.g., skin characteristics), establishing the model geometry, determining the connections between components, and setting appropriate boundary and loading conditions. After these, researchers applied FEA solver software for the solution phase and then identified the relevant results to serve the analysis objectives. Each step involves critical decisions that can significantly influence the simulation outcomes. Therefore, this review examines the diverse approaches taken in FEA studies on skin flap design, aiming to summarize and evaluate the key decisions made at each step of the FEA process. Additionally, new technologies such as MR and AR were explored as means to bridge the gap between engineering research and clinical practice, facilitating the illustration of results for clinicians and patients without an engineering background. In this context, the potential role of MR and AR in enabling patient-specific surgical planning was evaluated. An overview of the workflow for this review is presented in Figure 2.

### 2.1 Material modeling of skin

Skin is composed of a fibrous network rich in collagen, embedded within a ground substance matrix. Its mechanical behavior is nonlinear viscoelastic, exhibiting anisotropic characteristics depending on the orientation of the fibrous network. The mechanical properties of skin vary among individuals, influenced by factors such as gender and age, and change over time (Wijn et al., 1976; Lanir, 1981; Smith et al., 1982; Stark, 1977). It is worth noting that, in addition to these factors, the mechanical properties of skin also depend on the methods used to obtain the data. Various experimental techniques, including indentation, torsion, suction, uniaxial and dynamic testing, and optical methods, have been used to characterize human skin. Yazdi and Baqersad (2022) reviewed the literature studies published from 1969 to 2021, analyzing 130 papers focused on the study of mechanical properties of human skin. They summarized and compared the skin data obtained through different experimental methods. The reported mechanical properties varied significantly among studies. For example, the Young’s modulus of forearm skin can vary by more than 50,000-fold between its lowest and highest reported values. Using an *in vivo* indentation test, Bader and Bowker (1983) measured a Young’s modulus of 1.09 kPa in the forearm skin of a young female subject. In contrast, Grahame and Holt (1969) reported values ranging from 18 to 57 MPa using an *in vivo* suction experiment, highlighting how skin stiffness changes with aging. The Young’s modulus also varies depending on the location of the skin on the human body. For instance, a modulus of 83.3 MPa was reported for back skin (Yazdi and Baqersad, 2022; Ní Annaidh et al., 2012) through uniaxial tensile *ex vivo* tests. This value was significantly different from that measured in forearm skin by Bader and Bowker (1983). Additionally, it was also found that compared to normal skin, the scar tissue has increased stiffness (Dunn et al., 1985; Corr and Hart, 2013).

The large variances in Young’s modulus are attributed to experimental errors, the high heterogeneity of human skin, and its nonlinear mechanical behavior. This wide variability and uncertainty present a challenge for FEA in accurately predicting skin behavior for patient-specific skin flap design as documented values may differ significantly from those of an individual patient. To model skin mechanics, researchers have applied various approaches. Table 1 compares different skin modeling approaches used in FEA for flap design. Some studies simplified skin as an isotropic, linear elastic material, while others represented it as a nonlinear, isotropic, hyperelastic material, deriving parameters through experimental data fitting. Additionally, certain studies accounted for the orientation of collagen fibers, incorporating anisotropic properties. Other tissues (e.g., bone), although mentioned in a few papers such as Retel et al. (2001), were not included in this section as they are not directly relevant to the application of FEA in skin flap design.

**TABLE 1:** Skin flap material modeling.

Study	Constitutive law of skin modeling	Property value	Pros and cons of the approach
Retel et al. (2001)	Linear isotropic for elements in tension or compression in both x- and y-directions; linear orthotropic for elements, with the x-direction in tension and the y-direction in compression	Young’s modulus is 1 MPa in tension and 0.3 MPa in compression. Poisson’s ratio is 0.4	Pros: The skin model shows reduced stiffness in compression versus tension, with behavior resembling 3D skin tissue buckling above a threshold, although this study is 2D. Cons: Compression regions are manually identified using a criterion, but this is impractical for complex simulations where such manual selection is not feasible
Azar et al. (2001)	Piecewise linear isotropic	Young’s modulus is 3.43 MPa for strain between 0 and 0.54, 28.9 MPa for strain between 0.54 and 0.68, and 157 MPa for strain between 0.68 and 1. Poisson’s ratio is 0.49999	Pros: The model captures skin’s nonlinear behavior, where stiffness increases at high strains due to dominant collagen fiber effects. Cons: Nonlinear analysis requires more computational time than linear analysis in FEA
Miyamoto et al. (2010)	Linear isotropic	Young’s modulus is 0.1 MPa for skin and 5.0 MPa for scar. Poisson’s ratio is 0.4	Pros: This model is easy to implement in FEA with high computational efficiency. Cons: It is a simplified model that excludes nonlinear hyper-elasticity and anisotropic behavior of skin
Lapeer et al. (2010)	Three hyperelastic models, namely, general polynomial, reduced polynomial, and Ogden model, were used to fit stress–strain curves obtained from uniaxial tests; reduced polynomial models that fit the curves best were used in the FEA	Based on the stress–strain curve fitting, the maximum Young’s modulus is 14.309 MPa. Poisson’s ratio is 0.4995	Pros: The model is based on *in vitro* test curve fitting to capture the skin’s hyperelastic behavior. Cons: Nonlinear analysis requires more computational time than linear analysis in FEA. *In vitro* tensile results likely differ from *in vivo* outcomes
Pauchot et al. (2013)	Isotropic incompressible nonlinear elastic model using eight chain material model of rubber from Arruda and Boyce (1993)	Parameters were obtained from the publication of Bischoff et al. (2000). The initial modulus is 102 kPa	Pros: The model captures the skin’s nonlinear behavior using a rubber-like material model. Cons: Nonlinear analysis requires more computational time than linear analysis in FEA
Remache et al. (2015)	Nonlinear rubber material model developed by Ogden (1972), which is isotropic, incompressible, homogeneous, and hyperelastic	Parameters of the Ogden model were obtained by fitting uniaxial tensile tests on human skin	Pros: The model captures the skin’s nonlinear behavior using a rubber-like material model. Cons: Nonlinear analysis requires more computational time than linear analysis in FEA
Lee et al. (2019)	Gasser–Ogden–Holzapfel (GOH) model by Gasser et al. (2006), which is a hyperelastic anisotropic material model taking into account the dispersion of the collagen fiber orientation	Parameters of the model were obtained by fitting *in vitro* tensile test data from NíAnnaidh et al. (2012) and Tonge et al. (2013)	Pros: This model includes an anisotropic collagen fiber network within an isotropic matrix, reflecting the skin’s direction-dependent properties. Cons: The model is complex and challenging to apply in FEA with intricate geometry, where determining the orientation of collagen fibers may be difficult
Ji et al. (2024a)	Hyperelastic anisotropic GOH model	Skin material parameters were set to be uncertain	Pros: The skin material parameters were assumed to follow a normal distribution based on literature data. Anisotropic behavior was modeled by varying the fiber orientation in the skin. Cons: The model is complex and challenging to apply in FEA with intricate geometry
Guo et al. (2024)	Neo-Hookean model, which is a hyperelastic material model	Young’s modulus is 200 kPa. Poisson’s ratio is 0.48	Pros: The model captures the skin’s hyperelastic behavior. Cons: It is a simplified model
Chen et al. (2025)	Linear isotropic model	Young’s modulus is 0.12 MPa and 0.23 MPa for forearm and forehead skin of the young patient, respectively, and 0.26 MPa for the forearm skin of the elderly patient. Poisson’s ratio is 0.49	Pros: This model is easy to implement in FEA with high computational efficiency. Cons: It is a simplified model that excludes nonlinear hyper-elasticity and anisotropic behavior of skin

As stated previously, the complex microstructure of skin, coupled with significant variability in its mechanical properties across individuals and different anatomical locations, poses challenges for the numerical analysis of skin flaps. Furthermore, measurement techniques and subject-specific factors contribute to the difficulty of achieving accurate modeling. To navigate these complexities, researchers have introduced various assumptions and simplifications in skin flap material modeling, leading to diverse material models with distinct parameters as shown in Table 1. In essence, the skin flap properties used in FEA rely heavily on theoretical predictions found in the literature, each incorporating different aspects with unique assumptions. This variability in modeling approaches hinders the development of patient-specific flap designs, in contrast to the more predictable FEA outcomes observed in implant studies. Unlike engineering materials, which possess stable and well-defined properties that can be represented using straightforward material models, skin is a living tissue with highly dynamic mechanical responses. This makes personalized modeling particularly difficult.

Therefore, obtaining more accurate skin properties remains a key challenge for researchers and clinicians working in the field. As extracting a skin sample near a wound for mechanical testing to inform FEA might not be a practical option in clinical settings, the solution may lie in developing non-invasive techniques to accurately assess individual skin characteristics. Recent advancements also highlight the need for contact-free testing methods to evaluate the mechanical behavior of human skin. Various imaging modalities, such as optical coherence tomography (OCT), photoacoustic tomography (PAT), and reflectance confocal microscopy (RCM), have been used for skin analysis, offering promising approaches for non-invasive assessment (Lentsch et al., 2022).

Based on the findings, future research should focus on refining non-invasive techniques to obtain patient-specific mechanical property parameters before surgery, which can then serve as the input for FEA, as also reported by Myoung et al. (2021). With advancements in imaging technologies, such as high-speed cameras, obtaining skin parameters for surgical quantification is becoming increasingly feasible. For example, Myoung et al. (2021) introduced the Swing anglemeter, a device designed to assess skin elasticity. Their study involved 45 healthy Korean women aged 23–60 years, using a rubber ball dropped onto the subjects’ cheeks and tracking its rebound trajectory. A mobile phone camera was used to capture the maximum rebound angle for each test, which was then correlated with skin elasticity. Although this method only provides a general assessment of skin condition rather than direct mechanical parameters for numerical analysis, it suggests a promising path toward a non-invasive, user-friendly approach for property estimation before surgery, potentially enabling the quantification of flap surgery procedures.

### 2.2 Geometry, mesh, connection between different parts, and software

Imaging technologies such as axial computed tomography (CT) scans and magnetic resonance imaging (MRI) have been widely used in the medical field since the last century for diagnosis, surgical planning, and post-procedural follow-ups (Vannier and Marsh, 1996). Computer-assisted surface imaging techniques have been used in plastic and reconstructive surgery since the late 20th century (Marsh et al., 1986). In the 21st century, 3D scanning methods have become increasingly prevalent for surface scanning, enabling the collection of detailed surface data for plastic reconstruction. For example, the commercially available FaceSCAN 3D (3D-Shape GmbH, Erlangen, Germany) was used to capture 3D images, which were then applied to evaluate the aesthetic outcomes of nasal plastic reconstruction (Peters et al., 2021). With advancements in computational power and numerical analysis software during the 2000s, numerical simulations became both feasible and practical for assisting in plastic surgery procedures, including syndactyly reconstruction. These imaging techniques have since been integrated into the construction of geometric models for FEA, enhancing the precision and effectiveness of surgical planning and evaluation.

In the preprocessing stage of FEA of skin flap, researchers must make several critical decisions, including geometry construction, meshing strategies, defining connections between different parts of the model, and selecting software used for geometry creation, meshing, and solving. We reviewed these aspects based on selected studies and summarized them in Table 2 for comparison.

**TABLE 2:** Geometry, mesh, connection between different parts, and software.

Study	Geometry	Mesh, 2D or 3D behavior	Preprocessing (imaging and meshing) and FEA software
Retel et al. (2001)	The skin was modeled as a rectangular plate with a thickness of 1 mm. Skin wound was modeled as a diamond-shaped hole within the model	Plane stress quadrangular elements (PLANE 82) and triangular elements (TRIANGLE2)	ANSYS
Miyamoto et al. (2010)	3D images of the middle and ring fingers from CT images were converted into stereolithography (STL) format. Scar was modeled as a column, with a diameter of 1 mm	3D element (Solid 185)	DICOM manager (INTAGE); ANSYS
Pauchot et al. (2013)	Utilized predefined geometry to model a V–Y advancement flap	Plane stress triangular element	ANSYS
Remache et al. (2015)	Utilized predefined geometry to model a V–Y advancement flap	Plane stress triangular element (PLANE183)	ANSYS
Lee et al. (2019)	Three local flap designs, namely, advancement, transposition, and rotation flaps, were schematically created to illustrate the geometric definitions of each procedure. Each incision design was embedded within a planar tissue patch, with the skin defect represented as a circular shape. For the scalp, where the surgery was performed, an average thickness of 3.5 mm was assumed	3D eight-node linear brick element (C3D8)	ABAQUS
Ji et al. (2024a)	A handy SCAN 3D handheld laser scanner (accuracy: 0.03 mm) was used to capture wound surface data, which then unfolded to the flat surface to serve as a template. The completed geometric wound model was exported in .stp format. Skin flap with scaling ratios of 95%, 90%, 85%, and 80% were then imported into the FEA	Three-node universal shell element (S3)	ABAQUS
Guo et al. (2024)	A predefined circular skin area containing a rhomboid-shaped defect	A 3D tetrahedra mesh was generated from a 2D planar mesh to consider different layers of skin flap. The simulation continued running until it either converged or reached 40 iterations	Not mentioned in the paper
Chen et al. (2025)	Three predefined flap designs with a quadrangular flap, a triangular flap, and one based on a central axis	3D element (Solid 185); mesh convergence was achieved	ANSYS

Table 2 shows that some studies have analyzed the effect of skin flap parameters using predefined geometries in FEA. In these cases, no imaging conversion was required as the geometries could be easily generated using the software’s modeling functions. Miyamoto et al. (2010) constructed a 3D model based on CT scans; however, they did not simulate the suture closure process. Instead, they treated the skin flap and underlying soft tissue as a single solid object. Ji et al. (2024a) used a laser scanner to capture the wound surface data, obtaining the 3D geometry of the wound surface. They then applied an algorithm they had developed to unfold the 3D surface into a 2D representation (Ji et al., 2024b). Notably, their study explicitly stated the contact condition between the hand’s bone, skin tissue, and grafted skin flap as frictionless contact. This contrasts with other studies, where some researchers used a plane stress condition without considering the underlying structures.

Despite significant advancements in 3D imaging techniques, 3D surface scanning has not been widely applied in FEA for flap design. This may be due to the irregular shape of the human body surface, particularly in hand anatomy, making the numerical modeling of skin flap coverage and closure challenging. It is important to note that effectively integrating FEA into patient-specific flap design for complex surfaces and shapes requires iterative adjustments to achieve an optimal surgical plan. The numerical difficulty of such a 3D contact problem would make it challenging to obtain convergent results. Therefore, unfolding an irregular 3D surface into a 2D plane appears to be a promising approach, as demonstrated by Ji et al. (2024b). Given the complex contours of the human body—especially the hand—3D scanning, combined with computational techniques to flatten the surface, could greatly enhance feasibility. This method would facilitate flap geometry construction, improve mesh quality, reduce computation time, and simplify result visualization and analysis. When studying the stress and strain in a skin flap stretched over a wound, flattening the surface would also facilitate the application of boundary and loading conditions.

However, presenting the FEA results in 2D might not be intuitive to everyone, especially for surgeons, who are accustomed to working with 3D representations of anatomical structures during surgery. Future work could focus on developing methods to transition from 2D to 3D surface visualization. MR and AR technologies are well-suited for this task as they can provide real-time, interactive 3D visualizations that transform the 2D results into a more intuitive 3D representation, even restoring the original shape of the flattened surface. This would allow clinicians to view and interact with the flap design in its true anatomical form while still benefiting from the simplified 2D representation for analysis. There were proposed algorithms that deform or bend flat surfaces into 3D curved surfaces, which provide valuable insights into how 2D surfaces can be transformed into 3D shapes (Jin et al., 2022; Sam et al., 2024). Although these algorithms have not been specifically tested in MR and AR devices, these platforms possess the same computational capacity as regular computers, allowing for their implementation in real-time, interactive environments. This should be evaluated in future research.

### 2.3 Simulated procedures, boundary and loading conditions, and results

The skin flap surgery involves wound closure with suture stitches using various flaps. This procedure can be simulated by applying appropriate boundary and loading conditions during the preprocessing stage of FEA. Once the preprocessing is completed, FEA can be conducted. The FEA results (i.e., postprocessing variables) include force, displacement, stress, strain, and other variables. Therefore, the specific results to be considered and generated in a study must be carefully selected based on the objectives and requirements of the analysis. Table 3 summarizes the simulated procedures, boundary and loading conditions, and postprocessing variables applied in the studies. It shows that some researchers accounted for the pretension of the skin surrounding the wound before surgery by applying prestress to simulate this effect, while others did not. The boundary and loading conditions generally involve either fixation or displacement control to simulate the suture process for wound closure. Another observation is that, although not explicitly stated in the papers, all the analyses appear to be static. This is consistent with expectations as no dynamic behavior is considered in the simulation of wound closure. Some studies validated their FEA results by comparing them with clinical observations, theoretical models, or surrogate data—for example, by identifying consistency with known stress patterns in skin necrosis or flap surgery. Others did not include formal validation but still provided valuable insights through trend analysis, such as examining the influence of varying material parameters. These trend-focused studies remain meaningful despite the lack of validation as their goal was comparative analysis rather than precise clinical prediction.

**TABLE 3:** Simulated procedures, boundary and loading conditions, expected variables, and result validation in the studies.

Study	Simulated procedure	Boundary and loading condition	Postprocessing variable	Result validation
Retel et al. (2001)	A diamond defect covered by the Limberg flap	Boundary: edge of skin was fixed. Load: Displacement was applied to the edge of the wound	Von Mises stress and closure force	The result obtained using the material model with differing stiffnesses in compression and traction was compared to that from an isotropic linear elastic model, in which compression and traction stiffnesses are equal
Miyamoto et al. (2010)	Dorsal rectangle flap with different shaped tips for webspace reconstruction in syndactyly release surgery	Boundary: “The proximal edges of the middle and ring finger bones were fixed.” Load: “Displacements of 10 mm were applied to the middle phalanxes to mimic the hand-opening motion”	Von Mises stresses and web displacement	No validation was performed for the FEA results
Pauchot et al. (2013)	V–Y advancement flap to cover a rectangular defect	(Part I) Boundary: zero displacement on left and bottom sides of the skin sheet. Load: a biaxial prestress on right and top sides. (Part II) Boundary: zero displacement on a point of one side of the wound. Load: displacement on the other side to move the edge to mimic closure	Normal stress	The stress results from the FEA were compared with clinical cases of skin necrosis and epidermolysis to identify similarities and validate the findings
Remache et al. (2015)	V–Y advancement flap to cover a skin defect of rectangular shape	Boundary: a quarter of a rectangular sheet of skin was simulated, and the symmetry boundary condition was applied on the edges of the symmetry. Load: first, biaxial prestress was applied on the skin sheet. Second, displacement on a point of one side of the wound was set to 0, and the nearest point on the other side was moved to mimic closure	Closure force	No validation was performed for the FEA results
Lee et al. (2019)	Three types of local flap, namely, advancement, transposition, and rotation flaps, for a circular skin defect closure	Boundary: edge of skin was fixed. Load: applied displacement to close the flap by reducing the distance of the two pair of nodes	Von Mises stress and normal stress	FEA results were compared with those of the surrogates
Ji et al. (2024a)	A clinical patient’s hand wound flap covered by flaps with different fiber orientations and different sizes	Boundary: bone tissue and the edge of the skin were fixed. Load: skin flap connected the edge of the surrounding skin through connectors. Displacement was applied at the skin flap node on the one side of the connector to simulate the suturing closure process	Stress	FEA results were compared with those of the surrogates
Guo et al. (2024)	Rhomboid flap for closing a circular skin defect	Undermining region setup: one of the boundaries was fixed, and the undermining area was increased by displacing the other boundary to a certain value. Suture process: springs with zero rest-length were set between the two vertices. Increasing the spring stiffness reduced the distance, simulating the closure process	Suture force	No validation was performed for the FEA results
Chen et al. (2025)	Reading man flap based on different designs of angles and central axial lengths to cover a circular skin defect	Boundary: the edge of skin was fixed. Load: displacement was applied to the edge	Von Mises stress	FEA stress concentration locations matched those observed in the flap surgery

In summary, these studies primarily examined closure or suture forces and stresses within the skin flap. Although uncertainty in skin material properties impacts the reliability of the results, the findings still provide valuable insights for trend analysis, particularly when comparing the effects of geometric and material parameters.

### 2.4 MR and AR visualization techniques with FEA in surgery

There is a current trend of applying novel visualization approaches, such as MR and AR, to integrate FEA results into surgical workflows. Although other means, such as colormaps and software interfaces, exist, MR and AR offer enhanced real-time interaction and immersive visualization. This helps medical professionals better interpret engineering principles and results more effectively in surgical decision making.

A common use of MR and AR visualizations integrating FEA results in surgery is to provide real-time representations of organ or soft tissue deformation (Golse et al., 2021; Kong et al., 2017; Perruisseau-Carrier et al., 2017; Nikolaev and Cotin, 2020; Samei et al., 2018; Kugler et al., 2017). For example, AR systems have been developed and evaluated to assess the feasibility of dynamically updating liver deformation (Golse et al., 2021; Nikolaev and Cotin, 2020; Kugler et al., 2017). By integrating AR with FEA results, this approach allows the liver model in AR to align with the organ’s shape in real time during surgery. CT scans were usually obtained to reconstruct a 3D organ model preoperatively, which allows for precise anatomical segmentation and patient-specific surgical planning. By integrating an FEM, the reconstructed organ model can dynamically adapt to intraoperative deformations (Golse et al., 2021; Nikolaev and Cotin, 2020; Kugler et al., 2017). This ensures accurate alignment with real-time surgical conditions, thereby providing precise localization of tumors and resection planning (Nikolaev and Cotin, 2020), along with real-time surgical guidance (Kugler et al., 2017). A similar approach was used in a study that developed an AR-guided navigation system for precise intraoperative tumor localization during laparoscopic kidney surgery (Kong et al., 2017). The virtual organ model dynamically updates in real time to reflect deformations caused by surgical manipulation. Prostate deformation was also visualized in an AR application that integrated FEM with real-time transrectal ultrasound (TRUS) imaging to enhance intraoperative navigation during robot-assisted laparoscopic radical prostatectomy (RALRP) (Samei et al., 2018). This approach also improved tumor localization and surgical precision (Samei et al., 2018). In addition to organs, Perruisseau-Carrier et al. (2017) focused on applying patient-specific MRI data for the 3D reconstruction of nerve structures and then integrating FEA to simulate nerve deformation under physiological and surgical conditions. AR overlaid the updated FEM nerve model onto the surgical field, allowing surgeons to visualize real-time nerve deformations and adjust their approach accordingly.

There were also studies that have leveraged the capability of MR and AR to provide a sense of depth (Vörös et al., 2023; Tonutti et al., 2017). Vörös et al. (2023) investigated the use of a 3D autostereoscopic display in a simulated laparoscopic task to restore depth perception for surgeons. By integrating an MR simulator with an FEM, the system allowed participants to visualize soft tissue deformation in 3D and improve task performance compared to conventional 2D visualization. The results demonstrated significant reductions in task completion time, instrument travel distance, and error rates. This highlighted the benefits of depth-enhanced visualization in minimally invasive surgery. In addition, a machine learning approach was proposed to enhance visualization by allowing the surgeon to visualize internal structures with depth perception through an AR headset (Tonutti et al., 2017). By integrating pre-computed FEA results with artificial neural networks (ANNs) and support vector regression (SVR), the system accurately modeled soft tissue deformation under varying loads. This enabled real-time updates of tumor position with errors below 0.3 mm, demonstrating significant potential for assisting surgeons in more precise tumor localization and resection.

From the abovementioned descriptions, AR and MR can be regarded as wearable computing systems that enable real-time computation and optimization. Studies have also demonstrated their capability to integrate machine learning and deep learning algorithms to predict various surgical outcomes and anatomical changes dynamically. For example, a study applied AR to predict the final shape of a catheter during endovascular procedures by integrating fiber Bragg grating (FBG) sensors with FEA results (Scarponi et al., 2024). By leveraging machine learning algorithms, the system continuously refined predictions, enhancing surgical precision and reducing reliance on repeated fluoroscopic imaging. Machine learning algorithms have been used to accelerate FEA-based deformation predictions for real-time use (Samei et al., 2018), which is critical in intrasurgical applications.

## 3 Discussion

Recent FEA studies have increasingly addressed the complexity and variability of individual human anatomy to improve modeling precision and adaptability to patient-specific cases. For instance, Lee et al. (2019) incorporated the orientation of collagen fibers in the skin, while Ji et al. (2024a) modeled skin material parameters as normally distributed variables based on population data, accounting for natural variability. They also captured anisotropic behavior by adjusting fiber orientation in the skin model. Chen et al. (2025) further advanced personalization by comparing biomechanical differences between young and elderly patients. Despite recent advancements, several significant challenges persist. One major challenge is the high degree of uncertainty in skin properties and their sensitivity to anatomical location, which makes it difficult to achieve clinically viable, patient-specific skin flap optimization using FEA. To address this limitation, future research should focus on developing and refining non-invasive methods for accurately measuring patient-specific mechanical properties preoperatively, for example, the approach proposed by Myoung et al. (2021). These parameters could then inform more precise and individualized FEA models.

Another key challenge lies in creating a geometric model due to the complexity of the human body surface. Some efforts have focused on flat surfaces and regularly shaped wound models (Rajabi et al., 2015; Okamoto et al., 2018; Papadakis et al., 2023). Chen et al. (2025) reported the application of FEA in designing a reading man flap, which is commonly used for the closure of circular skin defects in craniofacial and plastic surgery. They studied eight cases with varying flap angles and central axis lengths to simulate the stress and strain changes within the skin after the flap transfer. The results indicated that an angle of 60° for quadrangular flaps and 45° for triangular flaps, combined with a central axis length twice the diameter of the circular defect, were optimal for flap design. They also found that flap tip stress in elderly patients was higher than that in younger patients when using the same design, potentially increasing the risk of vascular insufficiency and localized flap necrosis. Therefore, in clinical practice, flap designs may benefit from incorporating such FEA-based findings to reduce complications. Although these findings provide valuable design guidance, flap design in hand surgery presents additional challenges: it must not only cover complex 3D wound defects but also support functional restoration. The interaction between skin and irregular wound surfaces further complicates accurate simulation (Ji et al., 2024b). In this context, emerging 3D scanning technologies offer a promising path by accurately capturing wound surfaces and converting them into 2D representations to guide precise flap design and support functional restoration. This approach could support the development of preoperative planning tools tailored to individual patients.

Integrating novel, cutting-edge technology, such as MR and AR, has shown great potential in other medical fields, which holds promise for advancing skin flap design using FEA. This would assist in multiple aspects, including transitioning from 2D to 3D surface visualization and helping select the most suitable flap design before surgery. Following this, post-surgery evaluations, continuous feedback, and iterative refinement will be essential for improving the system. In the long term, AI-driven approaches represent the future direction for building a database to refine and validate the method. As data continue to grow with information gathered from diverse patients in the database, the FEA-assisted flap design is expected to become increasingly accurate, personalized, and adaptable, leading to improved preoperative decision-making and post-surgical outcomes. Although this has been scarcely studied in the context of skin flap design, similar applying AI-driven approaches, such as big data analytics, have been adopted in other medical fields for planning purposes. For example, De Momi et al. (2016) demonstrated the use of AI in robotic surgery systems to analyze large datasets for optimized trajectory planning in robot–human handover tasks. They trained an ANN using human action data to plan tool handovers to surgeons. The proposed trajectory planner was shown to improve robot–human psychophysical interaction during handover tasks. This research highlights the potential of AI-driven approaches to enhance real-time decision making and planning, which could similarly be applied to FEA-assisted flap design to personalize and optimize surgical strategies. One possibility is that by collecting individual skin mechanical properties, hand shape, wound shape, used flap, final surgical outcomes, and other relevant data to generate large datasets, the system could learn patterns and recommend the optimal flap size and shape prior to surgery, predicting the final outcome.

## 4 Conclusion

FEA is a powerful tool for designing skin flaps and predicting mechanical behavior during surgical procedures. The accuracy of FEA models relies heavily on factors such as skin mechanical properties, model geometry construction, and boundary and loading conditions. However, the complex, nonlinear, and highly variable mechanical properties of human skin present significant challenges for patient-specific modeling. Although the material models reviewed and summarized in this work can offer valuable insights for trend analysis, such as identifying optimal scar patterns, they are not yet capable of providing accurate predictions for individual patients. This limitation is a critical barrier to personalized FEA applications as skin property variability greatly influences model outcomes. Addressing this issue will require the development of non-invasive techniques to obtain individualized skin data. In terms of geometry and meshing, methods such as unfolding 3D wound surfaces into 2D representations can improve mesh quality and computational efficiency. In addition to this, although validations occurred in some studies, there remains a need for large-scale, multicenter efforts to clinically validate FEA simulation predictions and ensure their robustness and generalizability. Additionally, FEA results are generally intuitive for those with an engineering background, but the interpretation of the results poses a challenge in clinical practice. To bridge this gap, technologies such as AR and MR can be used to convert simulation outputs into real-time, interactive 3D visualizations, enhancing clinical usability. These visualization tools have already demonstrated success in areas like tumor localization and organ deformation tracking, supporting both preoperative planning and intraoperative accuracy. Although these methods have not yet been applied to flap design in hand surgery, insights from their use in other contexts can inform future developments in the field. It is also found that integrating machine learning with AR/MR platforms offers promising potential for real-time surgical prediction and guidance. In the long term, combining FEA with AI-driven approaches and expanding clinical datasets could enable adaptive, personalized flap design systems that significantly enhance surgical decision making and patient outcomes.

In summary, this review not only analyzed recent studies on the use of FEA in skin flap design but also identified a development pathway for patient-specific surgical planning. We recommend that future research focus on 1) advancing non-invasive techniques for acquiring patient-specific skin properties, 2) exploring technologies such as unfolding 3D wound surfaces into 2D representations to improve mesh quality and computational efficiency, 3) clinically validating FEA simulation predictions through large-scale, multicenter studies to ensure robustness and generalizability, 4) developing real-time AR/MR-assisted systems that seamlessly integrate simulation or optimization outputs into surgical workflows, and 5) creating AI-driven platforms capable of continuously learning from clinical data to deliver adaptive, personalized flap design recommendations. These advancements would enhance the precision, efficiency, and patient-centered nature of surgical outcomes.
